# The Interaction between Bmal1 and Per2 in Mouse BMSC Osteogenic Differentiation

**DOI:** 10.1155/2018/3407821

**Published:** 2018-03-29

**Authors:** Haiya Zhuo, Yuhong Wang, Qing Zhao

**Affiliations:** State Key Laboratory of Oral Diseases, Department of Orthodontics, West China Hospital of Stomatology, Sichuan University, Chengdu, China

## Abstract

The circadian clock is a system that controls endogenous time of organisms, and it regulates the physiology and behavior of bodies. The transcription factors Brain and Muscle ARNT-like Protein 1 (BMAL1) and Period2 (Per2) are components of the circadian clock, and they play vital roles in circadian clock function. Both Bmal1−/− mice and Per2−/− mice display obvious bone volume changes. In this study, we inhibited the expression of Bmal1 in bone marrow-derived mesenchymal stem cells (BMSCs) using a lentiviral vector harboring RNAi sequences, which increased the osteogenic differentiation capability of BMSCs. We also suppressed Per2 gene expression using an adenovirus vector harboring RNAi sequences, and similarly, the osteogenic differentiation ability of BMSCs was enhanced. Furthermore, when both Bmal1 and Per2 gene expression was suppressed in BMSCs by lentiviral and adenoviral interference, the osteogenic differentiation capability was stronger than that in BMSCs following single-gene inhibition. Our data support that both Bmal1 and Per2 play negative roles in BMSC osteogenic differentiation and that Bmal1 and Per2 have a synergistic effect on the osteogenic differentiation of BMSCs.

## 1. Introduction

Bone is one of the most active organs in organisms, and it remodels throughout life. Research shows that there are some signaling pathways involved in bone metabolism, and the circadian clock is among them [1]. Serious consequences may appear if the circadian rhythm has disordered, such as a decrease of the bone mineral density as well as an increased risk of patients developing osteoporosis [2, 3]. Genes that take part in mesenchymal stem cell and osteoblast differentiation and genes that participate in mineral deposition are all expressed rhythmically [4–6]. Bmal1 and Per2 are both important components of the molecular biological clock, and they are expressed in bone with 24-h periodicity [7]. Mice that are deficient in Bmal1 show an accelerated aging phenotype and have no orthotropic osteogenesis, and older mice that are deficient in Bmal1 have lower bone weights [8, 9]. Bmal1−/− mice occur with low bone mass [10], but another study showed that Bmal1−/− mice had elevated osteoblasts numbers and bone-formation parameters (MAR: mineral apposition rate; BFR: bone-formation rate), indicating that Bmal1 inhibits bone formation. Studies demonstrate that Per1−/−; Per2m/m, Per1−/−; Per2−/−, and Per2^Brdm1^mutant mice all exhibit high bone mass phenotypes [11, 12]. Thus, Bmal1 and Per2 play vital roles in the regulation of bone formation. Additionally, Bmal1 and Per2 are both the core components of the circadian clock autoregulatory feedback loop. Whether Bmal1 and Per2 have an interaction in the regulation of bone formation is still unknown.

To explore the specific roles of Bmal1 and Per2 in the regulation of bone formation and to determine whether Bmal1 and Per2 have an interaction in this process, we inhibited the expression of Bmal1 or/and Per2 in BMSCs and observed their ability to differentiate into osteoblasts. The data showed that the mRNA and protein expression of osteogenic differentiation markers (Alp, Runx2, and Ocn), the ALP activity, and the ability of calcified nodule formation of BMSCs were enhanced after Bmal1 inhibition, which indicated that Bmal1 has a negative role in the regulation of osteogenic differentiation in BMSCs. After the inhibition of Per2, the osteogenic differentiation capability of BMSCs was similarly enhanced, demonstrating that Per2 also has a negative role in the regulation of osteogenic differentiation in BMSCs. After the simultaneous suppression of Bmal1 and Per2, the osteogenic differentiation capability of BMSCs were much stronger than those in BMSCs following single-gene inhibition, which indicated that Bmal1 and Per2 have a synergistic effect on the osteogenic differentiation of BMSCs.

## 2. Materials and Methods

### 2.1. Cell Culture

BMSCs (passage 5) isolated from 4-week-old C57/BL6 male mice were purchased from the Cyagen Biosciences incorporated company (Guangzhou, China). The BMSCs were identified as CD29(+), CD44(+), CD117(−), and CD31(−), and passage 12 BMSCs had good adipogenesis and osteogenesis abilities, as described by the seller. The cells were cultured in a humidified atmosphere with 5% CO2 at 37°C and fresh alpha-minimum essential medium (*α*-MEM) containing 10% fetal bovine serum (FBS), 100 mg/ml streptomycin, and 100 U/ml penicillin (basic medium); the medium was changed 2-3 times per week. When BMSCs were 80%–90% confluent, they were passaged using 0.25% trypsin and 0.01% ethylenediamine-tetra acetic acid (EDTA). The passage 5 cells were used in the experiments below.

### 2.2. Experimental Groups

There were seven groups in the present study: the vacant control BMSC group (group 1, Vacant), the Bmal1 shRNA lentivirus vector-transfected BMSC group (group 2, Bmal1 repression group, Lenti-Bmal1 shRNA), the green fluorescent protein- (GFP-) expressing lentivirus vector-transfected BMSC group (group 3, Lenti-GFP-Puro), the Per2 shRNA adenovirus vector-transfected BMSC group (group 4, Per2 repression group, Adeno-Per2 shRNA), the red fluorescent protein- (RFP-) expressing adenovirus vector-transfected BMSC group (group 5, Adeno-RFP-Puro), the Bmal1 shRNA lentivirus- and Per2 shRNA adenovirus-transfected BMSC group (group 6, Bmal1 and Per2 repression group, Lenti-Bmal1 shRNA-Adeno-Per2 shRNA), and the GFP-expressing lentivirus and RFP-expressing adenovirus-transfected BMSC group (group 7, Lenti-GFP-Puro-Adeno-RFP-Puro). Regarding the last two groups, we first constructed stable Bmal1 shRNA lentivirus-transfected BMSC lines and GFP-expressing lentivirus-transfected BMSC lines and then added Per2 shRNA and RFP-expressing adenovirus, respectively.

### 2.3. The Construction of Stable Bmal1 shRNA-Transfected BMSC Lines and GFP-Expressing Lentivirus-Transfected BMSC Lines

The passage 5 BMSCs were seeded in 6-well plastic plates at an initial density of 5 × 10^5^ cells per well in basic media, and the Bmal1 shRNA lentivirus (Arntl shRNA, synthesized by Hanheng bio, Shanghai, China) (group 2, Bmal1 repression group, Lenti-Bmal1 shRNA) or the GPF-expressing lentivirus (pHBLV-U6-ZsGreen-Puro) (group 3, Lenti-GFP-Puro) was added per well at a multiplicity of infection (MOI) of 20. After 24 h of cell culture, the medium was replaced with the basic one, and 48-h later, the cells were screened by basic medium supplemented with 2 *μ*g/ml puromycin. The green fluorescence level was observed under an inverted fluorescence microscope (OLYMPUS IX70, Japan), and we calculated the transfection efficiency by RT-qPCR and Western blotting assays. The cells were passaged until they were stable, and after two passages, the adequate cells were cryopreserved for the following experiments.

### 2.4. Per2 shRNA Adenovirus Vector and RPF-Expressing Adenovirus Vector Transfection

The BMSCs, stable Bmal1 shRNA-transfected BMSCs and GFP-expressing lentivirus-transfected BMSC, were cultured in 24-well plastic plates at an initial density of 4 × 10^4^ cells per well in basic media, and after the cells were 80% confluent, the Per2 shRNA adenovirus vector (synthesized by Hanheng bio, Shanghai, China) was added to the wells containing BMSCs and Bmal1 shRNA-transfected BMSCs (group 4, Adeno-Per2 shRNA and group 6, Lenti-Bmal1 shRNA-Adeno-Per2 shRNA, resp.), and the RFP-expressing adenovirus vector (synthesized by Hanheng bio, Shanghai, China) was added to the wells containing BMSCs and GFP-expressing lentivirus-transfected BMSC (group 5, Adeno-RFP-Puro and group 7, Lenti-GFP-Puro-Adeno-RFP-Puro, resp.) at an MOI of 20. The fluorescence levels were observed under an inverted fluorescence microscope after 48 h, and we determined the transfection efficiency using Western blotting assays. The dilution of anti-BMAL1 (1 : 500), anti-PER2 (1 : 500) and anti-*β*-actin primary antibodies (1 : 1000) were used. And the dilution of horseradish peroxidase-conjugated secondary antibody (1 : 1000) was used.

### 2.5. RT-qPCR Analysis

The cells in those seven groups were cultured in 6-well plates with 1 × 10^5^ cells per well, and several groups were transfected by the corresponding adenovirus as required. After the cells were 80% confluent, they were cultured in the osteogenic medium (basic medium together with 50 *μ*g/ml L-ascorbate, 10 nM dexamethasone, and 10 mM *β*-glycerophosphate), and mRNA expression was evaluated after 7 and 14 days of osteoinduction. The cells were harvested and kept in an RNA preservation solution (RNA safeguard). The total RNA was extracted applying a simple P total RNA extraction kit. The absorbance (A) at 260 nm was measured using a spectrophotometer to quantify the total RNA, and an A260 : A280 ratio of 2.0 of RNA samples was considered to be of high purity. Two milligrams of total RNA acquired from each sample was used for reverse transcription applying the SYBR-Prime ScriptTM RT-PCR Kit following the manufacturer's protocol. The real-time PCR was carried out in a 20-*μ*l reaction mixture in triplicate on an ABI PRISM 7300 Real-time PCR system (Applied Biosystems, USA) according to the manufacturer's protocol. The housekeeping gene *β*-actin was used as the internal reference, and primers applied to real-time PCR analysis are shown in Table 1. We calculated the initial copy numbers of unknown samples using the 7300 Real-time PCR system from the standard curve.

### 2.6. Western Blotting

The cells in those seven groups were cultured in 6-well plates with 1 × 10^5^ cells per well, and some groups were transfected by the corresponding adenovirus as required. After cells were 80% confluent, they were cultured in the osteogenic medium as described in the RT-qPCR protocol, and total protein was obtained after 7 and 14 days. GAPDH (Glyceraldehyde-3-phosphate dehydrogenase) or *β*-actin were used as internal references. After being cultured for the abovementioned times, those cells were washed with ice-cold phosphate-buffered saline (PBS) twice and then lysed by lysis buffer from the Keygen total protein extraction kit. Then we collected the supernatant after the lysate was centrifuged at 14,000 *g* for 15 min at 4°C and the supernatant was quantitatively assayed using the BCA (bicinchoninic acid) method. We disposed total protein extracts using standard SDS-PAGE procedures and subsequently transferred them to a PVDF membrane. And after blocking, we probed the membranes using the appropriate specific primary antibodies and horseradish peroxidase- (HRP-) conjugated secondary antibodies. We observed the immunoreactive proteins applying a chemiluminescence kit. The ChemiDoc XRS Gel documentation system and Quantity One software (Bio-Rad) were used to determine the band intensities.

### 2.7. Quantitative Alkaline Phosphatase and Protein Assays

The cells in those seven groups were cultured in 6-well plates with 1 × 10^5^ cells per well, and some groups were transfected by the corresponding adenovirus as required. To quantitatively analyze alkaline phosphatase activity, PBS was used to wash the cells after osteoinduction for 7 days, and 10 mM Tris HCl and 0.1% Triton X-100 (pH 7.4) were used to lyse the cells. These cells were then processed by repeating the freeze-thaw cycle three times. After sonication, ALP activity was biochemically measured with a modified method of King applying a commercial kit as instructed. The total protein content was measured using the Bradford method; the reactions were evaluated at 510 nm and calculated based on bovine *γ* globulin standards. The ALP data are expressed as unit/L protein.

### 2.8. Alizarin Red Staining

The cells in those seven groups were cultured in 24-well plates with 4 × 104 cells per well, and some groups were transfected by the corresponding adenovirus as required. After cells were 80% confluent, they were cultured in the osteogenic medium as described in the RT-qPCR protocol. After 14 days of osteogenic induction, 4% paraformaldehyde was used to fix the cover glass from the experimental and control groups in 24-well plates for 20 min. After the cells were rinsed with PBS, they were stained with Alizarin red staining solution and incubated at room temperature for 30 min, and then the cells were observed under inverted phase microscope. The intensity of Alizarin red of each group was quantified by using the Image Pro-Plus 6.0.

### 2.9. Statistical Analysis

All experiments were conducted at least three times. Measurements are presented as the mean ± SD. Statistical comparisons were determined by factorial analysis of variance (ANOVA), followed by the Student–Newman–Keuls (SNK) test to do the multiple comparisons. Statistical significances were considered at a value of *p* < 0.05.

## 3. Results

### 3.1. Repression of Bmal1 and/or Per2 in mRNA and Protein Levels after Virus Transfection in BMSCs

**Figure 1:**
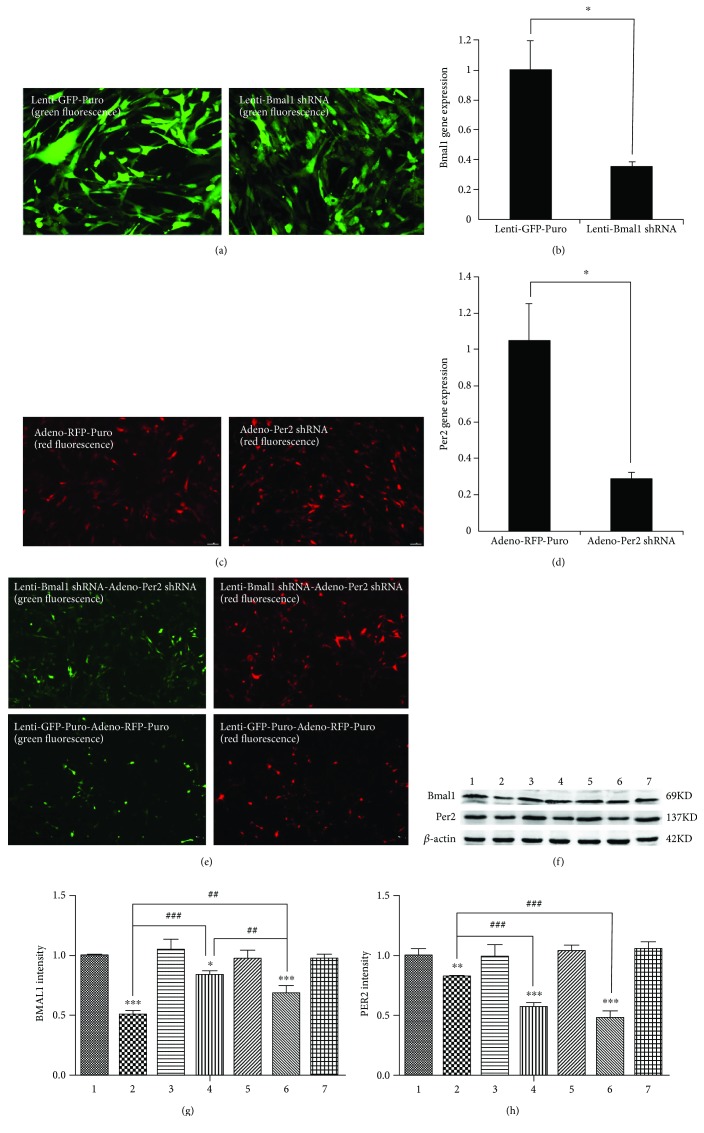
The decreased expression of Bmal1 and/or Per2 in mRNA and protein levels after virus transfection in BMSCs. (a) Expression of GFP in BMSCs infected by GFP-expressing lentivirus and Bmal1 shRNA lentivirus. After the BMSCs were infected for 48 h, we can observe abundant GFP. Magnification is 200x. (b) The expression level of Bmal1 mRNA 48 h after transfection. (c) Expression of RFP in BMSCs infected by RFP-expressing adenovirus and Per2 shRNA adenovirus. After the BMSCs were infected for 48 h, we can observe abundant expression of RFP. Magnification is 100x. (d) The expression level of Per2 mRNA 48 h after transfection. (e) Expression of GFP and RFP in the Bmal1 shRNA lentivirus- and Per2 shRNA adenovirus-transfected BMSC group (group 6, Lenti-Bmal1 shRNA-Adeno-Per2 shRNA) and the GFP-expressing lentivirus- and RFP-expressing adenovirus-transfected BMSC group (group 7, Lenti-GFP-Puro- Adeno- RFP-Puro). After the BMSCs were infected for 48 h, we can observe abundant expression of GFP and RPF. Magnification is 100x. (f–h) The expression level of BMAL1 and PER2 protein of BMSCs in each group 48 h after transfection. 1, 2, 3, 4, 5, 6, and 7 represented the Vacant, Lenti-Bmal1 shRNA, Lenti-GFP-Puro, Adeno-Per2 shRNA, Adeno-RFP-Puro, Lenti-Bmal1 shRNA-Adeno-Per2 shRNA, and Lenti-GFP-Puro-Adeno-RFP-Puro groups, respectively. The Bmal1 shRNA lentivirus (in group 2) weakened the expression of BMAL1 protein compared with the GFP-expressing lentivirus (in group 3), and it also downregulated the expression of PER2. The Per2 shRNA adenovirus (in group 4) weakened the expression of PER2 protein compared with the RFP-expressing adenovirus (in group 5), and it also downregulated the expression of BMAL1. In addition, the Bmal1 shRNA lentivirus and Per2 shRNA adenovirus (in group 6) weakened the expression of BMAL1 and PER2 protein compared with the GFP-expressing lentivirus and RFP-expressing adenovirus (in group 7). *β*-actin served as the endogenous reference gene. Densitometry by Quantity One software was used to quantify the protein level, and the protein level was displayed as the ratio of BMAL1 and PER2 relative to *β*-actin. Data represent the mean ± SD (*n* = 3); the asterisk indicates the group compared to the control groups, that is, group 1, group 3, group 5, or group 7. ^∗^*p* < 0.05, ^∗∗^*p* < 0.01, and ^∗∗∗^*p* < 0.001. The pound key indicates a significant difference between the two groups (^#^^#^*p* < 0.01, ^#^^#^^#^*p* < 0.001).

After 48 h of lentiviral infection, the inverted fluorescence microscope was used to detect the GFP in BMSCs (Figure 1(a)). As shown in Figure 1(b), the transfection efficiency was greater than 60%, and the suppression of Bmal1 mRNA transcripts was successful in the Bmal1 repression group. Inhibition of the Bmal1 gene was further confirmed at the protein level by Western blot analysis (Figures 1(f) and 1(g)). The expression of the BMAL1 protein in the Bmal1 repression group displayed an obvious decrease compared with the Lenti-GFP-Puro group. In addition, the expression of PER2 protein also decreased in the Bmal1 repression group.

After 48 h of adenoviral infection, RFP was detected in BMSCs under an inverted fluorescence microscope (Figure 1(c)). As shown in Figure 1(d), the transfection efficiency was greater than 70%, and suppression of the Per2 mRNA transcripts was successful in the Per2 repression group. Inhibition of the Per2 gene was further confirmed at the protein level by Western blot analysis (Figures 1(f) and 1(h)). In addition, the expression of BMAL1 protein also decreased in the Per2 repression group.

After 48 h of lentiviral and adenoviral infection, GFP and RFP were detected in BMSCs under an inverted fluorescence microscope (Figure 1(e)). The Bmal1 shRNA lentivirus and Per2 shRNA adenovirus (in group 6) weakened the expression of BMAL1 and PER2 protein compared with the GFP-expressing lentivirus and RFP-expressing adenovirus (in group 7) (Figures 1(f)–1(h)). Inhibition of Bmal1 and Per2 gene expression in all groups was confirmed at the protein level by Western blot analysis (Figures 1(f)–1(h)).

### 3.2. Expression of Osteogenic Differentiation Markers in Each Group after Osteoinduction

**Figure 2:**
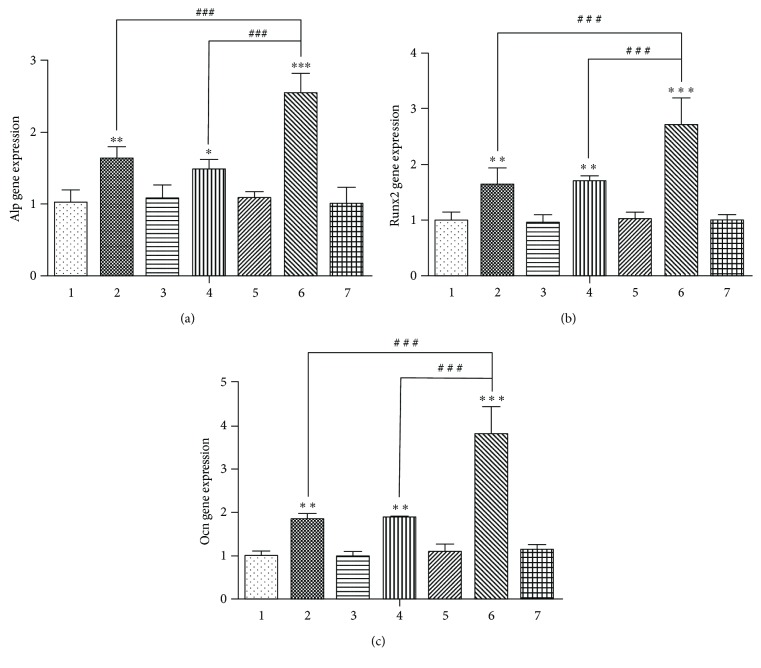
Alp, Runx2, and Ocn mRNA expression among the groups after 7 days of osteoinduction. (a) Alp mRNA expression. (b) Runx2 mRNA expression. (c) Ocn mRNA expression. Data represent the mean ± SD (*n* = 3); the asterisk indicates the group compared to the control groups, that is, group 1, group 3, group 5, or group 7. (^∗^*p* < 0.05, ^∗∗^*p* < 0.01, and ^∗∗∗^*p* < 0.001). The pound key indicates a significant difference between the two groups (^#^^#^^#^*p* < 0.001).

**Figure 3:**
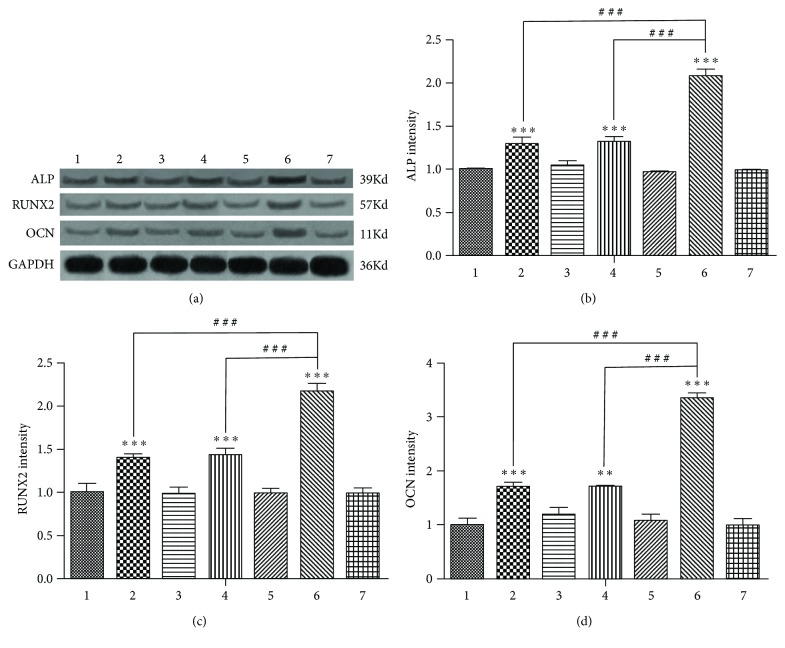
After 7 days of osteoinduction, Western blotting results showed the same trend of ALP, RUNX2, and OCN protein expression as the Bmal1, Runx2, and Ocn mRNA among the seven groups. GAPDH served as the endogenous reference gene. (a) The result of Western blotting. (b) The ALP protein expression intensity. (c) The RUNX2 protein expression intensity. (d) The OCN protein expression intensity. Densitometry was used to quantify the protein level; the protein level was displayed as the ratio of ALP, RUNX2, and OCN relative to GAPDH. Data represent the mean ± SD (*n* = 3); the asterisk indicates the group compared to the control groups, that is, group 1, group 3, group 5, or group 7. ^∗∗^*p* < 0.01 and ^∗∗∗^*p* < 0.001. The pound key indicates a significant difference between the two groups (^#^^#^^#^*p* < 0.001).

After 7 days of osteoinduction, there was no statistically significant difference in the mRNA expression of Alp, Runx2, and Ocn among group 1, group 3, group 5, and group 7 (Figure 2), indicating that GFP and/or RFP does not interfere with the expression of Alp, Runx2, and Ocn in the four vacant virus groups. Inhibition of the Bmal1 or Per2 genes (in group 2 and group 4) enhanced the mRNA expression of Alp, Runx2, and Ocn (Figures 2(a)–2(c)). However, after the simultaneous inhibition of Bmal1 and Per2, the mRNA expression of Alp, Runx2, and Ocn was much stronger than that in the single-gene suppression groups (Figures 2(a)–2(c)). The difference in the expression of BMAL1, RUNX2, and OCN protein level among the 7 groups was in accordance with the RT-qPCR results (Figures 3(a)–3(d)).

**Figure 4:**
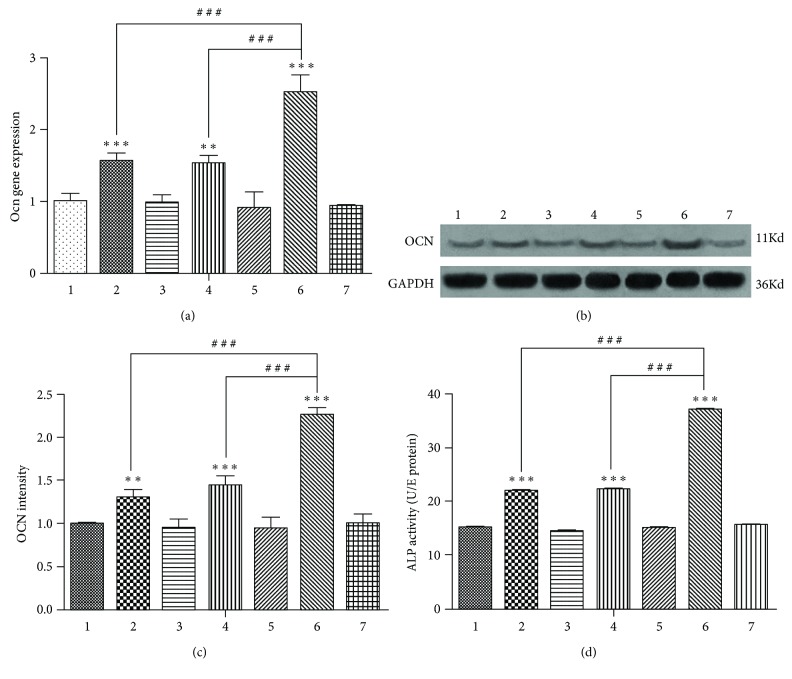
(a) Ocn mRNA expression among the groups after 14 days of osteoinduction. (b) Western blotting results showed the same trend of OCN protein expression as the Ocn mRNA among the seven groups after 14 days of osteoinduction. (c) The OCN protein expression intensity. GAPDH served as the endogenous reference gene. Densitometry was used to quantify the protein level; the protein level was displayed as the ratio of OCN relative to GAPDH. (d) Alkaline phosphatase (ALP) activity among the groups after 7 days of osteoinduction. Data represent the mean ± SD (*n* = 3); the asterisk indicates the group compared to the control groups, that is, group 1, group 3, group 5, or group 7. ^∗∗^*p* < 0.01 and ^∗∗∗^*p* < 0.001. The pound key indicates a significant difference between the two groups (^#^^#^^#^*p* < 0.001).

After 14 days of osteoinduction, the difference in the mRNA expression of the late osteogenic differentiation marker Ocn among the 7 groups was in accordance with that after 7 days of osteo-induction (Figure 4(a)). The difference in the expression of OCN protein among the 7 groups was in accordance with the RT-qPCR results (Figure 4(b) and 4(c)).

### 3.3. Alkaline Phosphatase Activity after 7 Days of Osteogenic Induction

After 7 days of osteogenic induction, inhibition of the Bmal1 or Per2 gene increased the activity of the early osteogenic differentiation marker ALP (Figure 4(d)); after simultaneously inhibiting Bmal1 and Per2, the activity of ALP was much stronger than that in the single-gene suppression groups.

### 3.4. Bone Nodule Formation after 14 Days of Osteogenic Induction

**Figure 5:**
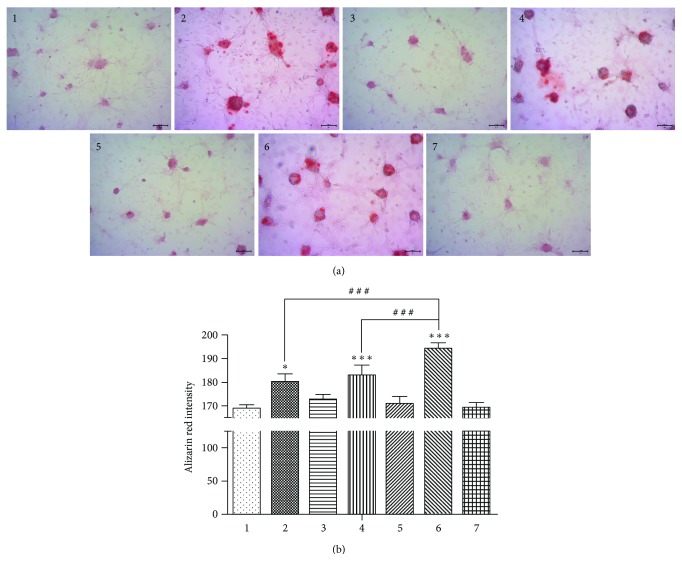
(a) Bone nodule formation after 14 days of osteogenic induction. The calcified nodules of group 2 and group 4 were more than that of the control groups, that is, group 1, group 3, group 5, or group 7. But the calcified nodule formation of group 6 was better than that of group 2 and group 4. Magnification is 100x. (b) The intensity of Alizarin Red among the groups after 14 days of osteoinduction. Data represent the mean ± SD (*n* = 3); the asterisk indicates the group compared to the control groups, that is, group 1, group 3, group 5, or group 7. ^∗^*p* < 0.05 and ^∗∗∗^*p* < 0.001. The pound key indicates a significant difference between the two groups (^#^^#^^#^*p* < 0.001).

After 14 days of osteogenic induction, inhibition of the Bmal1 or Per2 gene increased the formation of calcified nodules (Figure 5). However, after simultaneously inhibiting Bmal1 and Per2, the ability of calcified nodule formation of BMSCs was much stronger than that in the single-gene suppression groups.

## 4. Discussion

The present study showed that the mRNA and protein expression of osteogenic differentiation markers (Alp, Runx2, and Ocn), the ALP activity, and the ability of calcified nodule formation in BMSCs were enhanced after the inhibition of Bmal1 and Per2, demonstrating that the osteogenic differentiation capability of BMSCs was increased, and Bmal1 and Per2 have negative roles in the regulation of BMSC osteogenic differentiation. After simultaneously inhibiting Bmal1 and Per2, the osteogenic differentiation ability of BMSCs were much stronger than those following single-gene inhibition, indicating that Bmal1 and Per2 may have a synergistic effect on BMSC osteogenic differentiation.

### 4.1. The Relationship between Bmal1 and Bone Metabolism

Fu et al. demonstrated that Bmal1−/− mice had increased osteoblast numbers and bone-formation parameters, indicating that Bmal1 had a negative role in the regulation of bone formation [11]. Takarada et al. also showed that in 8-week-old Bmal1−/− mice, the osteoblast surface/bone surface, bone formation rate/bone surface and mineral apposition rate of the femur by histomorphometric analysis increased significantly [13]. Similarly, our data suggest that after the inhibition of Bmal1, the osteogenic differentiation capability of BMSCs was enhanced, indicating that Bmal1 has a negative role in the regulation of bone formation. However, Bmal1−/− mice did not exhibit a high bone mass (HBM), which may be explained by hypogonadism, a condition that increases bone resorption [14–17]. Urinary elimination of deoxypyridinoline crosslinks, a by-product of collagen degradation, was increased in Bmal1−/− mice, indicating that bone resorption was indeed enhanced in these mice, explaining, at least in part, the absence of the high bone mass phenotype in Bmal1−/− mice. Fujihara et al. showed that osteoclast-related genes, such as nuclear factor of activated T-cells, cytoplasmic 1 (NFATc1), and cathepsin K (CTSK), showed circadian rhythmicity similar to that of clock genes, such as Bmal1, Per1, and Per2, in the femur (cancellous bone) of mice [7]. A chromatin immunoprecipitation (ChIP) assay revealed the interaction between BMAL1 proteins and the promoter regions of NFATc1 and CTSK. Thus, the circadian clock gene Bmal1 affects not only bone formation metabolism but also bone resorption metabolism.

Samsa et al. found that Bmal1−/− mice had a low bone weight phenotype, exhibiting a decrease in bone mineral density as well as a reduction of the active osteocyte and osteoblast quantity; the ability of differentiation into osteoblasts of BMSCs decreased, which was different from the results of Fu et al. and our results. However, Fu et al. did their experiments mostly on 2-month-old mice, whose skeleton has not reached the peak of bone mass [18]; the mice were still young, were less affected by gene knockout and thus did not display signs of premature aging. In the study of Samsa et al., they did their experiments mostly on 5-month-old mice, when the growth of the skeleton is almost complete; therefore, the mice were more affected by premature aging symptoms caused by gene knockout. Consequently, the low bone mass phenotype of Bmal1−/− mice reported by Samsa et al. was probably because of the premature aging symptoms caused by Bmal1 knockout, which induced bone mass loss, and potential increase in bone formation caused by Bmal1 knockout were obscured by the impact of premature aging symptom-induced bone mass loss.

Our previous study and the research of Li et al. demonstrated that Bmal1 may regulate bone formation via the Wnt/*β*-catenin pathway and that glycogen synthase kinase-3*β* (GSK-3*β*) may act as the medium and link between Bmal1 and the Wnt/*β*-catenin pathway [19, 20]. Min et al. showed that Bmal1 may promote osteogenic differentiation by regulating the expression of BMP2 in MC3T3-E1 cells [21]. In regard to the relationship between Bmal1 and bone formation, their results seem to be different from those of this present study. However, the use of models that do not take into account the cellular context, differences in cell preparations or use of nonphysiologic loss- and gain-of-function approaches contributed to the observation of contrasting data. In most cases, there is a lack of environmental signals in vitro experiments, whereas rich environmental signals can be found in vivo. Consequently, for the reliability and practicability of the experimental results, we will perform in vivo experiments in the next step.

### 4.2. The Relationship between Per2 and Bone Metabolism

Maronde et al. demonstrated that compared with wild-type mice, the Per2^Brdm1^ mutant mice exhibited increased bone formation rate and bone volume at 3, 12, and 48 weeks old. In addition, osteoblast isolation from Per2^Brdm1^ mutant mice had a significantly greater capability to form calcium nodules after 14 days of osteogenic culture [12]. In agreement, our data showed that after inhibition of Per2, the osteogenic differentiation capability of BMSCs was elevated, indicating that Per2 has a negative role in the regulation of bone formation. This present study demonstrated that the ability of osteogenic differentiation in BMSCs was similarly enhanced after the inhibition of the Bmal1 or Per2 gene, whereas Per2^Brdm1^ mutant mice displayed a high bone mass phenotype, which was different from the phenotype of Bmal1−/− mice. A possible explanation is that Per2-deficient mice have milder premature aging symptoms than Bmal1-deficient mice. And thus, the mice were less affected by the premature aging symptom-induced loss of bone mass. Without the inhibitive effect of Per2 on bone formation, the mice displayed increased bone mass.

Fu et al. found that Per1−/−; Per2m/m and wild-type mice exhibited similar urinary elimination of deoxypyridinoline crosslinks, indicating that bone resorption was not obviously affected in these mice [11]. Maronde et al. discovered that both Per2^Brdm1^ mutant mice and mice deficient in Cry2 showed obviously incremental bone volume at 12 weeks of age, but Per2^Brdm1^ mutant mice displayed changes in parameters especial for osteoblasts and displayed no change of the osteoclast activity marker TRAP5b in the circulating serum. However, mice deficient in Cry2 displayed changes in parameters especial for osteoclasts, which decrease the serum levels of circulating TRAP5b without affecting the osteoblast activity markers. Therefore, Per2^Brdm1^ mutant mice increase their bone mass mainly through enhancing bone synthesis, and the circadian gene Per2 affects bone metabolism mainly by regulating bone formation, whereas it does not overtly affect bone resorption.

Regarding the Per2 signaling pathway in the regulation of bone synthesis, studies have found that Per1−/−; Per2m/m, Per1−/−; Per2−/−, and Cry1−/−; Cry2−/− mice all exhibit high bone mass phenotypes, indicating that bone formation is negatively regulated by the molecular clock [11]. Studies found that, while bone-formation parameters and bone mass decreased in wild-type mice after long-term leptin i.c.v. infusion, this treatment consistently enhanced bone-formation parameters in Per1−/−; Per2m/m mice. Their data support that the circadian gene Per mediates the leptin-dependent sympathetic inhibition of bone formation in osteoblasts and that the possible mechanism was via the downregulation of *c-myc* expression by Per, which inhibits the expression of *G1 cyclin*, subsequently extending of the cell cycle and inhibiting osteoblast proliferation.

### 4.3. The Correlations between Bmal1 and Per2 in the Regulation of Bone Metabolism

The present research showed that Bmal1 and Per2 had negative roles in the regulation of osteogenic differentiation in BMSCs and that Bmal1 and Per2 may have a synergistic effect on BMSC osteogenic differentiation. Several scholars have demonstrated that there is a correlation between Bmal1 and Per2 in terms of their activity and gene expression [22]. The expression of Per1 and Per2 is significantly decreased in all tissues of Bmal1−/− mice, and Bmal1 is also distinctly downregulated in tissues of Per1−/−; Per2−/− mice. In agreement, our data showed that after inhibition of Bmal1, the expression of PER2 protein was downregulated. In turn, after Per2 was inhibited, the BMAL1 protein expression was also downregulated. It explains, at least in part, the synergistic effect of Bmal1 and Per2 on the osteogenic differentiation of BMSCs.

Output signals from the suprachiasmatic nuclei (SCN) are believed to transmit standard circadian time to peripheral tissues through humoral routes and the sympathetic nervous system [23, 24]. Some papers have indicated that the sympathetic nervous system mediates the expression of core clock genes and regulates bone remodeling [25]. Hirai et al. demonstrated that isoproterenol (Iso) of MC3T3-E1 osteoblastic cells, a nonselective *β*-AR agonist, orchestrated the rhythmic expression of the canonical clock genes Bmal1 and Per2 [26]. Iso also made the mRNA of prostaglandin endoperoxide synthase 2 (Ptgs2, also known as Cox2, an important regulation factor of bone metabolism) express rhythmically. They found that the interaction between Bmal1 and Per2 mediated the rhythmic impacts of Iso on the expression of Ptgs2 in osteoblasts. In addition, after continuous Iso treatment, Ptgs2 was significantly decreased in bone. They used a bioinformatics approach to reveal that the promoter of Ptgs2 gene contained E-box and D-box sequences, demonstrating that the circadian oscillators regulated Ptgs2 gene in osteoblasts. Other studies have shown that the promoter of Per2 contains D- and E-box-binding elements [27–29]. Their results revealed that *β*-AR signaling regulate the expression of Bmal1 and Per2 in osteoblasts and that the BMAL1 and PER2 proteins control the expression of Ptgs2 by binding to the E-box of the Ptgs2 promoter, thereby controlling bone metabolism. There is indeed some correlation between Bmal1 and Per2 in bone metabolism regulation, and they can control this complex physiological activity through several shared signaling pathways.

## 5. Conclusion

Our results suggest that the osteogenic differentiation capability of BMSCs is enhanced after the inhibition of Bmal1 or Per2, indicating that Bmal1 and Per2 have vital negative roles in the regulation of BMSC osteogenic differentiation. Moreover, Bmal1 and Per2 may have a synergistic effect on the osteogenic differentiation of BMSCs.

## Figures and Tables

**Table 1:** The primers used for RT-qPCR analysis. ALP: alkaline phosphatase; Runx2: runt-related transcription factor 2; and Ocn: osteocalcin.

Target gene	Primers	Sequence	Fragment size (bp)
ALP	Forward primer	5′- ATAACGAGATGCCACCAGAGG-3′	140 bp
	Reverse primer	5′- TTCCACATCAGTTCTGTTCTTCG-3′	

RUNX2	Forward primer	5′- CTACCCAGCCACCTTTACCTAC-3′	190 bp
	Reverse primer	5′- GAACTGATAGGATGCTGACGAAG-3′	

OCN	Forward primer	5′- AGGAGGGCAATAAGGTAGTGAAC-3′	147 bp
	Reverse primer	5′- AGGCGGTCTTCAAGCCATAC-3′	

*β*-actin	Forward primer	5′- GAGACCTTCAACACCCCAGC-3'	263 bp
	Reverse primer	5′- ATGTCACGCACGATTTCCC-3′	
